# Poorly controlled type 2 diabetes is accompanied by significant morphological and ultrastructural changes in both erythrocytes and in thrombin-generated fibrin: implications for diagnostics

**DOI:** 10.1186/s12933-015-0192-5

**Published:** 2015-03-08

**Authors:** Etheresia Pretorius, Janette Bester, Natasha Vermeulen, Sajee Alummoottil, Prashilla Soma, Antoinette V Buys, Douglas B Kell

**Affiliations:** Department of Physiology, Faculty of Health Sciences, University of Pretoria, Private Bag x323, Arcadia, 0007 South Africa; Unit of Microscopy and Microanalysis, University of Pretoria, Pretoria, South Africa; School of Chemistry and The Manchester Institute of Biotechnology, The University of Manchester, 131, Princess Street, Manchester, M1 7DN Lancs UK

**Keywords:** Type II diabetes, Erythrocytes, Deferoxamine, Deferasirox

## Abstract

We have noted in previous work, in a variety of inflammatory diseases, where iron dysregulation occurs, a strong tendency for erythrocytes to lose their normal discoid shape and to adopt a skewed morphology (as judged by their axial ratios in the light microscope and by their ultrastructure in the SEM). Similarly, the polymerization of fibrinogen, as induced *in vitro* by added thrombin, leads not to the common ‘spaghetti-like’ structures but to dense matted deposits. Type 2 diabetes is a known inflammatory disease. In the present work, we found that the axial ratio of the erythrocytes of poorly controlled (as suggested by increased HbA1c levels) type 2 diabetics was significantly increased, and that their fibrin morphologies were again highly aberrant. As judged by scanning electron microscopy and in the atomic force microscope, these could be reversed, to some degree, by the addition of the iron chelators deferoxamine (DFO) or deferasirox (DFX). As well as their demonstrated diagnostic significance, these morphological indicators may have prognostic value.

## Introduction

Type II diabetes mellitus causes an ever-increasing burden on health care [1-4]. The prevalence for all age-groups worldwide was estimated to be 2.8% in 2000 and predicted to increase to 4.4% in 2030 [5]. Among adults in the US, the prevalence of undiagnosed diabetes is currently 4.1% and prediabetes a staggering 35.6% [6]. Type II diabetes is associated with three main glycaemic disorders: chronic hyperglycaemia; glycaemic variability; and iatrogenic hypoglycaemia [7], and also (frequently) comorbidities including dyslipidemia (high cholesterol levels) [8-11] and hypertension [12,13]. It has also been suggested that with disturbed lipid metabolism, lipid dysregulation precedes the hyperglycemia and increased insulin resistance is found in type II diabetes [14-17]. All of the comorbidities are potentially responsible for the cardiovascular and other complications [18,19]. Compared with individuals without diabetes, patients with type II diabetes have a considerably higher risk of cardiovascular morbidity and mortality [12,20-26]. The following paragraphs will briefly discuss systemic inflammation, RBC structure and fibrin clot structure in type II diabetes.

**Figure 1:**
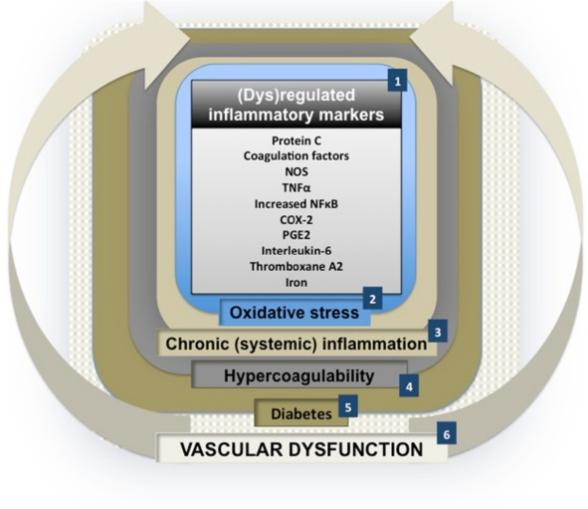
**Suggested chronological events in type II diabetes.** Inflammatory markers that are known to be dysregulated in diabetes (1); and to cause oxidative stress (2); resulting in chronic (systemic) inflammation (3); that have been associated with hypercoagulability (4). Dysregulations, as indicated by 1 to 4 have all been found in diabetes type II (5); 1 to 5 are early indicators of vascular dysfunction (6). Literature references are given in Table 1.

Diabetes is associated with (low-grade) systemic inflammation [27-29]. Table 1 inflammatory markers and their respective levels, that increased or decreased in diabetes. Suggested chronological events leading to vascular dysfunction in type II diabetes are summarized in Figure 1. We know that there are important links between oxidative stress, a changed inflammatory marker profile (inflammation), the development of diabetes type II as well as, ultimately, vascular dysfunction:

- Inflammatory markers are associated with systemic oxidative stress.
- Dysregulated iron levels are known to cause oxidative stress [30-34].
- Type II diabetes and oxidative stress is closely associated with chronic inflammation [35].
- Individuals with diabetes (and cardiovascular disease) demonstrate hypercoagulability [36,37] and hypercoagulability is key to inflammation [38].
- Ultimately, changed inflammatory marker levels, hypercoagulability and chronic inflammation, serve as early indicators of vascular dysfunction [39-41] see Table 1.

**Table 1:** **Selected references showing a changed inflammatory marker profile in diabetes type II individuals**

**Inflammatory marker**	**Selected references**
Low Protein C	[36,42]
High levels of coagulation factors (II, V, VIII, X and von Willebrandt factor)	[36,37,43-45]
Increased NOS	[46-50]
Increased TNFα	[25,51-53]
Increased NFκB	[28,54-57]
Increased COX-2	[57-61]
Increased PGE2	[62,63]
Iron (increased serum ferritin levels)	[64-89].
Increased interleukin-6	[44,45,53,62,90]
Increased thromboxane A2	[91,92]

Erythrocytes (RBCs) and atypical fibrin fiber formation or an altered fibrin structure are particularly common in inflammation [93-98]. Also, abnormalities of high blood glucose in the context of insulin resistance, and relative insulin deficiency, may disturb the architecture and functions of RBCs at molecular scale [99]. Membrane cholesterol has also been shown to alter the fluidity and bending stiffness of RBCs [100,101]. Previous studies suggest that RBC rheology is also altered in type II diabetes [102,103] and that hyperglycaemia has multiple effects on RBCs, including glycation of haemoglobin, reduced deformability and reduced lifespan [104]. It was also shown that an improvement in erythrocyte deformability from type II diabetes correlates with an improved glycaemic control [105]. Recently, we have also shown that RBCs in type II diabetes have a changed shape, as well as a decreased membrane roughness [93,94,106] and that RBCs can rapidly adapt in a changed environment, including during an addition of glucose to healthy RBCs [107].

Similar to changes in RBC, fibrin levels and thrombin generation are also changed, and it was indicated that there is enhanced thrombin generation and formation of denser fibrin clots of reduced lysability in type II diabetes [108]. A denser clot structure therefore results both from this changed fibrin concentration and thrombin generation [95,96].

Because type II diabetes is associated with dyslipidemia and hypertension, and most patients use medication to treat these co-morbidities; the medications typically prescribed are shown in Table 2. Here we also note evidence that suggests changes that the medication might have on RBC or fibrin structure.

**Table 2:** **Medication typically administered to diabetes type II patients and possible effects on erythrocytes (RBCs) and fibrin clots**

**Medication**	**Selected references**
**Dyslipidemia (cholesterol) medication (statins)**	
Simvastatin (Zocor**®**) and Atorvastatin (Lipitor**®**)	Improve clot permeability and clot structure and enhanced fibrin clot lysis [109-113].
	An increase in glycolysis metabolite concentrations and glucose-6-phosphate dehydrogenase activity in rat RBCs [114].
	Increased erythrocyte fluidity [115,116] and deformability [100,117].
	Reversed alteration in RBC plasma membrane properties, including lipid peroxidation [101,118].
**Blood sugar control**	
**Antihyperglycemic drug dimethylbiguanide** (Metformin**®**/Glucophage**®**)	Improves clot structure and hypercoagulability [119,120],
	Improves clot lyses [121,122].
**Actraphane®** (mixture of fast-acting insulin and long-acting insulin)	No evidence of any influence on RBCs or fibrin clot structure/fibrinolysis.
**Actrapid®** (human soluble insulin)	
**Humulin®** (70% human insulin isophane suspension and 30% human insulin injection [rDNA origin])	
**Protophane®** (intermediate-acting insulin)	
**Hypertension control**	
**Coversyl®** (active ingredient is perindopril arginine which is a angiotensin converting enzyme (ACE) inhibitor)	No evidence of any influence on RBCs or fibrin clot structure/fibrinolysis.
**Amlodopine®** (calcium channel blockers)	
**Carvedilol®** (beta and alpha adrenoceptor blocker with antioxidant activity)	Improves the endothelial fibrinolytic activity [123].
	Scavenger effect on free radical generator-induced RBC membrane damage [124] and enhances antioxidant defense mechanisms in RBCs [125].
**Adalat®** ((nifedipine) calcium channel blocker)	Antithrombotic activity exhibitor [126] and improves fibrinolytic activity [127,128].
**Anti-clotting medication**	
**Aspirin®** (acetylsalicylic acid)	Aspirin increases fibrin clot porosity and susceptibility to lysis [111]; antiplatelet effect [129-131];
**Disprin®** (brand name for Aspirin)	increase the level of sphingosine-1-phosphate and ceramide in erythrocytes [129] perturbing RBC bilayer structures [132].
	Reduce risk of thrombosis [133-137].
	Irreversible inhibitor of both cyclooxygenase COX-1 and COX-2 [138].

We have previously shown that there are profound changes in the hematological, and in particular, the erythrocyte and coagulation system in various inflammatory conditions where iron levels are increased (particularly serum ferritin levels) (see [139-144]). In view of this, in the current work, we studied the clot structure and erythrocyte structure of patients with type II diabetes. In addition, we also investigated whether two iron chelators, deferoxamine mesylate (DFO) and deferasirox (DFX) have an effect on the RBC and fibrin clot structure.

## Materials and methods

### Type II diabetes and healthy individual profiles

Ethical clearance was obtained from the Health Sciences Ethical Committee of the University of Pretoria. Healthy individuals were screened and chosen to participate in the study if they did not have any chronic condition, did not smoke or if female, use any hormone replacement or contraception. Diabetic individuals were chosen randomly from the diabetic clinic at the Steve Biko Academic Hospital. The patients were diagnosed according to the SEMSDA guidelines (http://www.semdsa.org.za/images/2012_SEMDSA_Guideline_July_FINAL.pdf). These guidelines follow the American Diabetes Association (ADA) criteria (classification and diagnostic criteria for diabetes were proposed by the American Diabetes Association) to define type II diabetes [145,146]. Citrated blood was collected for morphology studies and full iron profiles tests were done on both healthy and diabetic individuals. Plasma iron levels, HbA1c (Hemoglobin Alc) and medication were noted for each of the diabetes individuals (discussed later in detail in tables).

### Chelator addition to blood samples

Microscopy techniques were done with blood from diabetic patients with and without the addition of deferoxamine (DFO) and deferasirox (DFX), and with and without the addition of thrombin (final concentration chelator in whole blood were 3.33 mM and where thrombin was added, was 2.5 mM). To ensure a significant effect, we added an excess of each chelator; stoichiometric principles will ensure the full chelation of any unliganded iron present in whatever form. The solvent for both the chelators was DD H_2_O. Chelators were added to whole blood (WB) and left to react for 3 minutes. The kinetics of the chelators are virtually instantaneous and the potential for sequestration of the chelators by e.g. albumin is precisely why we added an excess of these reagents. When thrombin is added an extensive fibrin network is created around trapped RBCs. These WB preparation methods were done for all microscopy techniques (described in the following paragraphs).

### Light microscopy of erythrocytes

LM was used to study the axial ratios of RBCs, using 100 × magnification (with and without DFX and DFO). 10ul of WB was used to make a thin smear on a microscopic glass slide, this smear was left for 24 hours to air dry followed by fixing for 5 minutes in 100% methanol and left to air dry for 30 minutes. The smears were stained again for 4 minutes with Löffler’s methylene blue, and rinsed under running water followed by air-drying for 30 minutes. The final staining step involved staining for 30 seconds in Eosin Y-solution 0.5% aqueous, and rinsing with running water. Slides were viewed using a Nikon Optiphod transmitted light microscope.

### Axial ratio determination of erythrocyte shape

Axial ratios were determined from the LM micrographs, with the use of a program written in the C# programming language. The longest axis from each RBC was determined, referred to as the major axis, after which a perpendicular line was drawn in the centre of the major axis to establish the minor axis length. The axial ratio for each cell was obtained by dividing the major axis length by the minor axis length; a value of 1 represents a perfect circle.

### Scanning electron microscopy (SEM) of erythrocytes

High magnification SEM analyses were used to look at RBC structure and membrane surface. 20 μL of the fixed WB was dropped on a small glass coverslip to make smears, dehydrated, dried, mounted and coated with carbon according to previously described methods [93]. A Zeiss ULTRA Plus FEG-SEM with InLens capabilities was used to study the surface morphology of erythrocytes, and micrographs were taken at 1 kV.

### Atomic force microscopy of erythrocytes

Sample preparation was done according to previously described methods [143]. Characterization of RBCs was performed with a commercial AFM system (Dimension Icon with ScanAsyst, Bruker, USA) using the PeakForce QNM (Quantitative Nanomechanical Property Mapping) imaging mode [147]. At every pixel point a rapid force-distance curve is performed and as the cantilever’s deflection sensitivity and spring constant is calibrated before measurements, the curve can be analysed quantitatively to obtain a series of specific property maps of the sample. A retract curve is used to calculate modulus and adhesion images (slope of the curve and the minimum of the curve respectively) [141,143,148], the variation between the zero and maximum force is used to calculate deformation and the area between the approach and retract curve can be used to calculate energy dissipation [149]. The slope of the curve was fit, using the Derjaguin–Muller–Toporov (DMT) Model to determine the Young’s modulus (a measure of the stiffness of an elastic material) [141,150]. Silicon Nitride probes (TAP525 – MPP 13120–10, Bruker, USA) with a nominal force constant of 200 N.m-1, a resonant frequency between 430 and 516 kHz (measured by the manufacturer), and a nominal tip radius of 15 nm were employed in all AFM measurements.

From the diabetes sample, RBCs from the first 22 diabetic patients were analysed using AFM analysis, before and after exposure to the 2 chelators. Ten cells from each sample were analysed by selecting a 1 μm by 1 μm scan area on the periphery of the RBC and performing 128 by 128 data points of individual force curve measurements with a peak force of 6 μN. The periphery of the cells was chosen as not to be affected by the possible differences in concavity of the RBCs. The scans were performed at 0.6 Hz, which translates to a tip velocity of 1.2 μm/s and 50 force curves were chosen randomly within the stated area. Offline software (NanoScope Analysis version R3, Bruker, USA) was used to process the force curves and fit the modulus model to the unloading portion of the curve. The goodness of fit (R2) between the modulus model and the data given by the acquired curve is determined by calculating the ratio of explained variation to total variation in the dataset; only force curves with a goodness of fit so defined of 0.85 and above were used for modulus measurements. The statistical significance of the difference between calculations was determined using one-way analysis of variance. A P-value of less than 0.05 relative to the null hypothesis was considered to be ‘significant’ (cf. [151]), P-values are noted and boxplots drawn, together with descriptive statistics. A box plot was calculated using MS-Excel, together with the add-in template downloadable from http://www.vertex42.com/.

### Scanning electron microscopy of fibrin networks

10 μL platelet rich plasma (PRP) was mixed with 10 μL human thrombin provided by the South African Blood services. Extensive fibrin fiber networks were created and smears washed and fixed in 4% formaldehyde. Smears were prepared as described for RBCs and also viewed using Zeiss ULTRA Plus FEG-SEM with InLens capabilities. Fibrin fiber thickness were measured with ImageJ (ImageJ is a public domain, Java-based image processing program developed at the National Institutes of Health: http://rsbweb.nih.gov/ij/).

## Results

This study used 25 age- and gender-controlled healthy individuals (see Table 3) and 69 individuals with type II diabetes. The average age of healthy individuals was 55 (SD ± 11), while the average age of the diabetic individuals was 59 (SD ± 11). The current randomly selected type II diabetic population, in addition to their anti-diabetic medication, are on medication for dyslipidemia and hypertension as well (see Table 4). Despite their anti-diabetic medications, very few individuals showed HbA1c (Hemoglobin Alc) within the normal ranges, suggesting poorly controlled diabetes. In some patients, there were also abnormal iron levels (Table 4).

**Table 3:** **Demographic data from healthy individuals**

**Sample number**	**Gender**	**Age**	**Iron (μmol.L** ^**−1**^ **) 11.6-31.3**	**T Transferrin (g.L** ^**−1**^ **) 2.2-3.7**	**% saturation 20 -50%**	**Serum ferritin (ng.mL** ^**-1)**^ **M = 20-250 F = 10-120**	**Average axial ratio**	**± SD for axial ratio**
1	F	45	16.8	3	22	13	1.15	0.08
2	M	61	32.5	2.6	50	48	1.07	0.05
3	F	55	20	2.4	33	64	1.12	0.09
4	F	56	19.5	3.1	25	101	1.16	0.10
5	F	40	***10***	2.8	14	21	1.09	0.06
6	F	56	12.8	2.6	20	65	1.07	0.06
7	M	58	***10.2***	2.7	***15***	208	1.09	0.06
8	F	48	17.2	2.8	25	37	1.11	0.06
9	F	55	25.1	2.5	40	**121**	1.13	0.14
10	F	52	14.8	**2.2**	27	83	1.08	0.05
11	F	45	***31.6***	2.7	47	111	1.20	0.16
12	F	40	9.6	2.9	***13***	45	1.11	0.09
13	F	80	7.6	2.7	11	21	1.09	0.07
14	M	61	24.9	2.5	40	44	1.19	0.25
15	M	51	***10.8***	2.8	***15***	140	1.10	0.08
16	M	70	17	2.9	23	198	1.32	0.24
17	M	66	29.5	2.8	42	72	1.10	0.10
18	M	61	***7***	2.2	***13***	233	1.16	0.19
19	M	56	16.2	1.7	38	138	1.14	0.12
20	F	58	13.6	2.4	23	95	1.11	0.09
21	F	51	24.2	3.3	29	26	1.13	0.12
22	F	58	15.9	2.4	27	65	1.12	0.09
23	F	27	21.2	2.6	33	28	1.14	0.09
24	M	75	14.9	2.3	26	**393**	1.19	0.19
25	F	56	20.2	2.9	28	**159**	1.12	0.13

Bold values are above and italic values below the reference (normal) range.

**Table 4:** **Demographics, iron, HbA1c levels and medication usage of Type II diabetes patients**

**Sample number**	**Gender**	**Age**	**Iron (μmol.L** ^**−1**^ **) 11.6-31.3**	**Transferrin (g.L** ^**−1**^ **) 2.2-3.7**	**% saturation 20 -50%**	**Serum ferritin (ng.mL** ^**-1)**^ **M = 20-250 F = 10-120**	**HbA1c (%) <7%**	**Dyslipidemia: Simvastatin Atorvastatin**	**Met-formin/Oral**	**Actrapid Humulin Protoph-ane Actra-phane**	**HT: Coversyl Amlodopine Carvedilol Adalat**	**Aspirin/Disprin**
1	M	68	25.5	3.6	28	67		x	x		x	x
2	M	56	**49**	3	**65**	86	**11.9**		x		x	
3	M	71	28.1	2.2	**51**	210	**8.3**		x		x	
4	M	80	15.5	2.6	24	96	7	x	x		x	x
5	M	37	18.5	2.8	26	191	**7.6**		x			
6	M	56	***10.6***	2.7	***16***	39	**7.7**		x		x	
7	F	71	19.9	2.3	35	20	**8**	x	x		x	x
8	F	48	15.1	3.3	***18***	46	6.2	x	x		x	x
9	F	70	***5.2***	**3.9**	***5***	***7***	**7.5**		x		x	
10	F	82	**33.6**	3	45	101	**8.9**	x	x		x	x
11	M	62	25.6	2.3	45	202	5.9		x		x	
12	M	71	17.8	2.4	30	243	**10.5**	x	x		x	x
13	M	70	19.7	2.2	36	75	**10.4**		x		x	
14	F	58	**36.5**	2.9	50	**164**	6.0		x			
15	F	61	18.6	2.8	27	29	**8.2**		x		x	
16	M	56	**32.1**	2.9	44	152	**8.6**	x	x		x	x
17	M	42	**40.7**	2.3	**71**	217	**13.6**		x			
18	F	62	16.2	**4.1**	***16***	24	**10.6**	x	x		x	x
19	M	72	19.8	***2.1***	38	**332**	6.7		x	x	x	x
20	M	59	18.5	2.8	26	191	***?***	x	x			
21	M	75	14	***1.7***	33	218	6.6		x	x	x	x
22	M	41	28.2	3	38	235	**8.3**	x	x	x		x
23	F	81	23.8	2.3	41	62	**9**	x		x	x	x
24	M	41	**33.4**	2.7	49	101	5.5	x	x	x		x
25	M	80	**39.5**	2.7	**59**	142	6.8	x	x		x	
26	M	64	13.2	2.3	23	50	?		x	x		
27	M	63	22.9	2.4	38	**265**	**10.2**	x	x	x	x	x
28	F	49	**7.1**	3.4	***8***	12	6.8	x	x	x	x	
29	M	72	25.3	2.4	42	**386**	6.7		x	x	x	x
30	M	46	21	2.4	35	233	**11.6**		x			
31	M	40	14.5	2.4	24	96	**8.7**			x	x	
32	M	55	**10.3**	2.2	***19***	88	5.8		x	x	x	
33	F	62	BLOOD CLOTTED				**8.2**	x			x	
34	M	52	25.9	3	35	155			x		x	
35	F	59	18.5	2.5	30	**125**	**11.6**	x		x	x	
36	F	58	16.5	2.5	26	79	6.0	x	x	x	x	x
37	F	62	14.1	2.9	***19***	64	**10.2**	x	x	x	x	
38	M	60	***6.1***	2.6	***9***	93	**7.5**	x	x	x	x	x
39	F	57	24	2.7	36	60	**8.0**	x	x	x	x	x
40	F	63	31.4	2.5	50	77	**7.6**	x	x	x	x	
41	M	59	15.4	2.8	22	151	**8.5**	x		x	x	x
42	M	73	29.5	2.9	**51**	154	**8.0**	x		x	x	
43	F	58	20.3	***2***	41	81	**10.6**	x	x	x	x	
44	F	66	28.6	2.6	44	**359**	**10.0**	x	x	x	x	
45	M	60	18	2.4	30	192	**12.2**	x	x	x	x	
46	F	69	***9.6***	2.3	***17***	74	**7.4**	x	x	x	x	
47	M	62	21.1	3.1	27	28	**9.2**	x	x		x	
48	M	58	28.2	2.8	40	**605**	**11.9**	x	x	x	x	x
49	F	62	12.4	2.2	23	58	**11.3**	x	x		x	x
50	M	61	23	***1.9***	48	**1097**	6.9	x		x	x	x
51	M	73	**54.9**	2.4	92	100		x	x		x	x
52	F	45	***8***	3.7	***9***	***9***	**11.6**	x		x	x	x
53	F	62	**42.3**	2.4	**71**	**198**	**8**		x		x	
54	F	53	BLOOD CLOTTED				**10.9**	x		x	x	x
55	F	55	13.1	2.2	24	**189**	6.3	x	x	x	x	x
56	M	56	27	2.6	42	64	**12.1**	x	x	x	x	
57	F	70	17.8	3	24	23	**11.6**	x		x	x	x
58	F	59	**34.1**	2.7	**51**	56	5.8	x	x		x	x
59	M	66	**32.3**	2.7	48	123	**11.9**	x	x	x	x	x
60	F	54	BLOOD CLOTTED				**15.1**	x		x	x	
61	M	65	19.3	2.8	28	57	7.0	x		x	x	
62	F	56	16.6	2.6	26	24	**11.1**	x	x	x	x	
63	F	59	20.4	3.6	23	**149**						
64	F	64	***7.3***	**2.3**	***13***	82	**9.0**			x	x	x
65	M	32	16.8	3.2	21	58						
66	M	52	**129.6 checked**	3.2	**>95**	55	**8.1**	x	x	x	x	x
67	M	52	14.5	2.3	25	151	**12.2**	x		x	x	
68	F	49	14.2	2.7	21	17	**13.5**		x		x	
69	F	42	***6***	3.4	***7***	***9***	**8.3**	x	x	x	x	

Normal (healthy values) is given in the heading. Bold values are above and italic values below the reference (normal) range.

### Light microscopy

**Figure 2:**
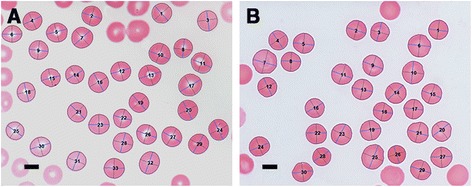
**Blood smears from two healthy individuals, showing axial ratio positions used for calculations. A)** Male: 60; serum ferritin: 48 ng.mL^−1^ **B)** male: 58; serum ferritin: 208 ng.mL^−1^. Scale bar = 5 μM.

**Figure 3:**
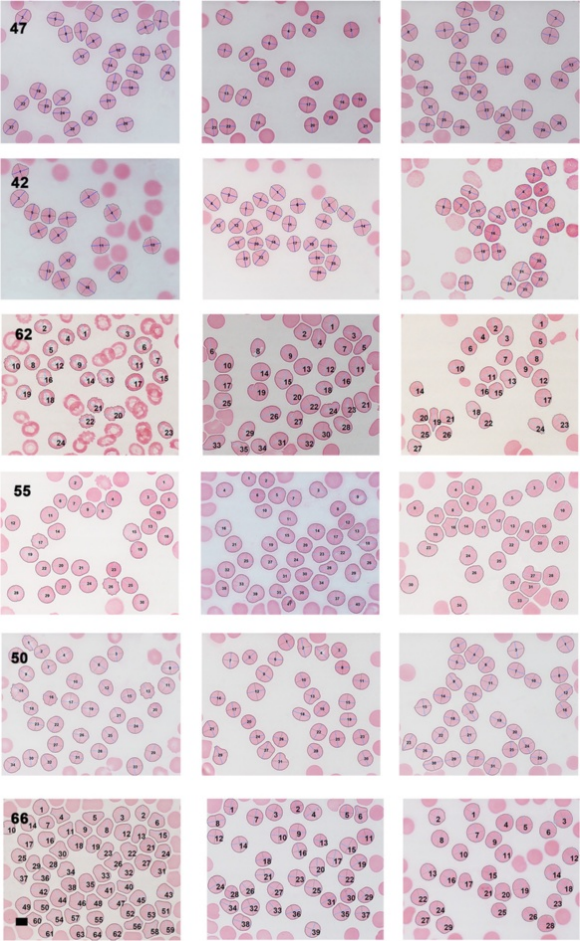
**Light microscopy smears of diabetic patients before and after treatment.** Left: untreated; middle: treated with deferasirox (DFX) and right: treated with deferoxamine (DFO). Patient detail is shown in Table 6. Scale bar = 5 μM.

**Figure 4:**
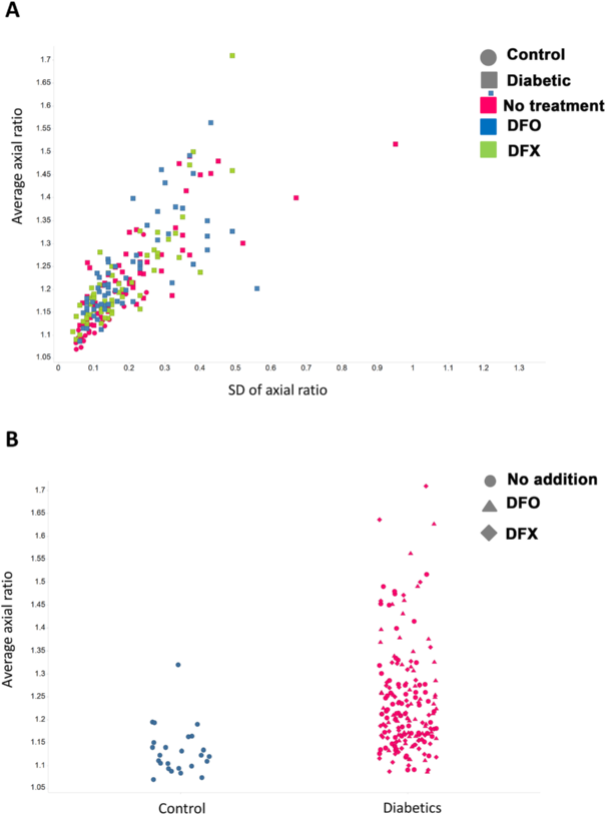
**Axial ratios of the erythrocytes from controls and diabetics.** As indicated in Table 5, there were significant differences in the axial ratios of diabetes vs controls, whether iron chelators were present or absent. **A**. Relationship between axial ratio and its standard deviation. **B**. Direct comparison between patients and controls. In **B** the values on the abscissa (only) are ‘jittered’ to make them easier to see.

Light microscopy of smears from typical healthy individuals, with their axial ratio positions indicated, is shown in Figure 2. Light microscopy statistics of the average RBC axial ratios of within group analysis are shown in Table 5. Figure 3 shows smears from diabetes patients before and after treatment with the 2 chelators (left: untreated diabetes; middle: DFX; right: DFO) Smears were chosen that represents the sample, e.g. normal iron levels, increased iron levels and whether addition of chelators showed a significant P-value (or not) (see Table 6); for specific details of the patients shown in Figure 3. A relationship between the RBC axial ratios and SD for the healthy controls, untreated diabetes and treated with DES and DEF was noted, and is illustrated in scatter plots (see Figure 4A and B).

**Table 5:** **Average axial ratios of the groups and significant differences between the groups**

	**Healthy individuals (no products added)** **(N = 25)**	**Untreated Diabetes (DIAB)** **(N = 69)**	**Diabetes with Deferasirox (DFX)** **(N = 69)**	**Diabetes with Deferoxamine (DFO)** **(N = 69)**	
**Average age and ± SD**	55 ± 11	59 ± 11			
**Cells analysed** **(** **N)**	3265	4365	4271	4369	
**Axial ratio average and ± SD**	1.14 ± 0.15	1.25 ± 0.27	1.24 ± 0.26	1.27 ± 0.27	
**SIGNIFICANT DIFFERENCE BETWEEN GROUPS (p-values shown)**					
**Healthy/Diab**	**Diab/DFX**	**Diab/DFO**	**DFX/DFO**	**Healthy/DFX**	**Healthy/DFO**
1.1E-56	4.7E-02	2.6E-04	2.6E-04	2.9E-83	2.0E-02

Data provided for healthy individuals compared with untreated diabetes, diabetes treated with deferasirox (DFX) and deferoxamine (DFO). Untreated diabetes results were also compared (comparison done on a paired basis) with diabetes treated with DFX and DFO.

**Table 6:** **Diabetes patient detail for light microscopy axial ratio micrographs, shown in Figure** **3**

	**AVERAGE AXIAL RATIOS**	**P1**	**P2**	**Gender**	**Age**	**Iron ((μmol.L** ^**−1**^ **) 11.6-31.3**	**Transferrin** **(g.L** ^**−1**^ **) 2.2-3.7**	**% saturation 20 -50%**	**Serum ferritin (ng.mL** ^**−1**^ **) M = 20-250 F = 10-120**
**DIABETES 47**	1.24	0.006	0.009	M	62	21.1	3.1	27	28
**DFX**	1.15								
**DFO**	1.17								
**DIABETES 42**	1.14	0.05	0.28	M	73	29.5	2.9	**51**	154
**DFX**	1.14								
**DFO**	1.17								
**DIABETES 62**	1.24	0.25	4.2 × 10^−7^	F	56	16.6	2.6	26	24
**DFX**	1.28								
**DFO**	1.45								
**DIABETES 55**	1.16	0.035	0.001	F	55	13.1	2.2	24	**189**
**DFX**	1.21								
**DFO**	1.25								
**DIABETES 50**	1.18	0.035	0.73	M	61	23	***1.9***	48	**1097**
**DFX**	1.14								
**DFO**	1.17								
**DIABETES 66**	1.33	5.8 × 10^−7^	3.9 × 10^−7^	M	52	**129.6**	3.2	**>95**	55
**DFX**	1.15								
**DFO**	1.11								

P-values: P1 (axial ratios of untreated diabetes RBCs versus DFX-treated) and P2 (axial ratios of untreated diabetes RBCs versus DFO-treated) (significant p-value was taken as ≤ 0.05; comparisons done on a paired basis) (Figure 3). Bold values are above and italic values below the reference (normal) range.

### Scanning electron microscopy (SEM) of erythrocytes

**Figure 5:**
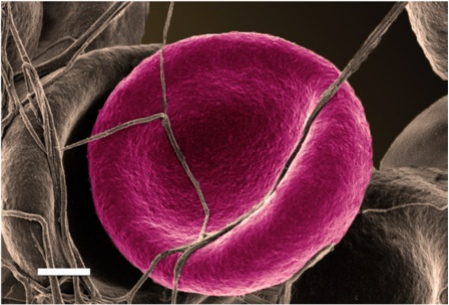
**Whole blood of healthy individual, with added thrombin, to create an extensive fibrin network around RBCs.** Male: 61; serum ferritin: 48 ng.mL^−1^. Scale Bar = 1 μM.

**Figure 6:**
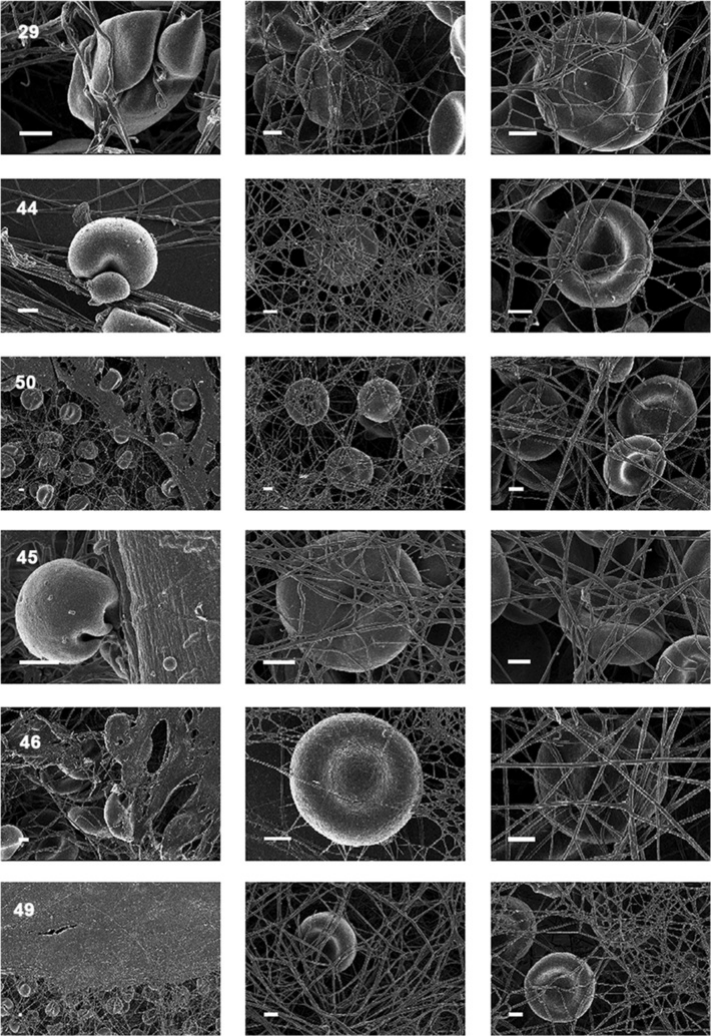
**Whole blood from diabetic patients before and after treatment.** Left: untreated; middle: Deferasirox (DFX) and right: deferoxamine (DFO). Table 7 shows the detail of the diabetic patients (sample numbers of diabetic patients shown on micrographs) shown in this figure. Scale Bar = 1 μM.

Figure 5 shows whole blood with added thrombin, which serves to create an extensive fibrin network around RBCs from a typical healthy individual. Figure 6 shows whole blood from diabetic patients before and after treatment where left, shows untreated WB, middle, shows WB treated with DFX and right, shows whole blood treated with DFO. Table 7 shows the detail of the diabetic patients shown in Figure 6. WB with added thrombin of healthy individuals forms a fibre net around the typical discoid RBCs. The RBCs keep their characteristic shape. However, in the diabetic populations, the RBC structure is compromised and in all the samples, the RBCs twist and fold easily in the presence of the fibrin fibres In the presence of the 2 chelators, it seems as if the RBCs do regain their typical discoid shape. See middle and right column micrographs of Figure 6. SEM and LM axial ratio analyses both showed that RBC shapes are changed in untreated RBCs of diabetic individuals (left column), but that chelator treatment seems to stabilize RBC shape and membranes (middle and right columns) (also noted in the AFM results shown in later paragraphs).

**Table 7:** **Diabetic patient details of micrographs from the SEM analysis shown in Figure** 6

	**Gender**	**Age**	**Iron (μmol.L** ^**−1**^ **) 11.6-31.3**	**Transferrin (g.L** ^**−1**^ **) 2.2-3.7**	**% saturation 20 – 50%**	**Serum ferritin (ng.mL** ^**−1**^ **) M = 20-250 F = 10-120**
**DIABETES 29**	M	72	25.3	2.4	42	**386**
**DIABETES 44**	F	66	28.6	2.6	44	**359**
**DIABETES 50**	M	61	23	**1.9**	48	**1097**
**DIABETES 45**	Yes	M	60	18	2.4	88
**DIABETES 46**	F	69	**9.6**	2.3	**17**	74
**DIABETES 49**	F	62	12.4	2.2	23	58

Bold values are above and italic values below the reference (normal) range.

### Atomic force microscopy of erythrocyte membranes

**Figure 7:**
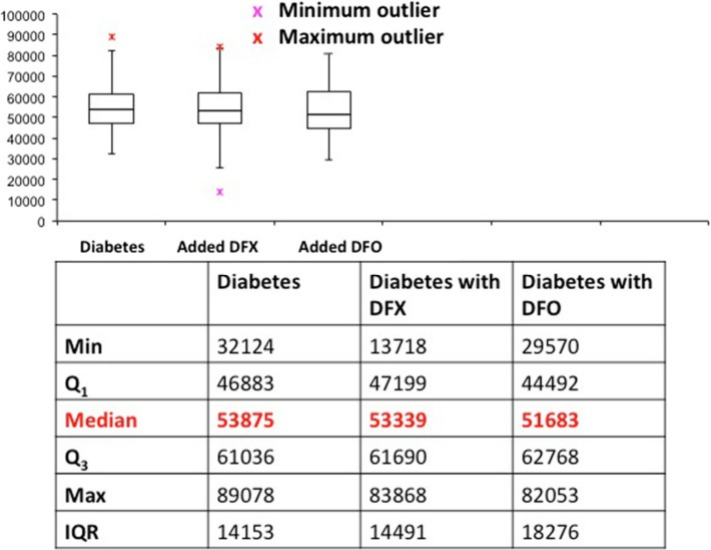
**Boxplot showing the variation of the Young’s modulus values of erythrocyte membrane stiffness, after the treatment of whole blood of diabetics with iron-chelating agents.** Young’s modulus median values did not vary significantly between the diabetes and two chelating agents (indicated in red in the figure).

A significant increase in the Young’s modulus (stiffness) was seen in the RBCs of 22 randomly selected diabetic patients, whose blood was treated with the 2 chelators. This reflects a decrease in elasticity and an assumed altered functionality in these cells. Treatment with either DFX or DFO decreased the Young’s modulus values towards more normal values, indicating a possible improvement in the elasticity of the cells (see Figure 7 and Table 8).

**Table 8:** **Statistical analyses of membrane stiffness as measured by Young’s modulus values of the diabetic group and treated groups**

**Group**	**Mean Young’s modulus**	**SD**	**P-value**
**Healthy individuals**	46710	39210	-
**Diabetes**	56483	64418	1.15E-13
**Diabetes + DFX**	51238	58225	2.7E-06
**Diabetes + DFO**	50446	36073	1.6E-10

(Diabetes p-value is from a pairwise comparison with the healthy individual group. DFX and DFO diabetes-treated p-values are from pairwise comparisons with the diabetic group).

**Figure 8:**
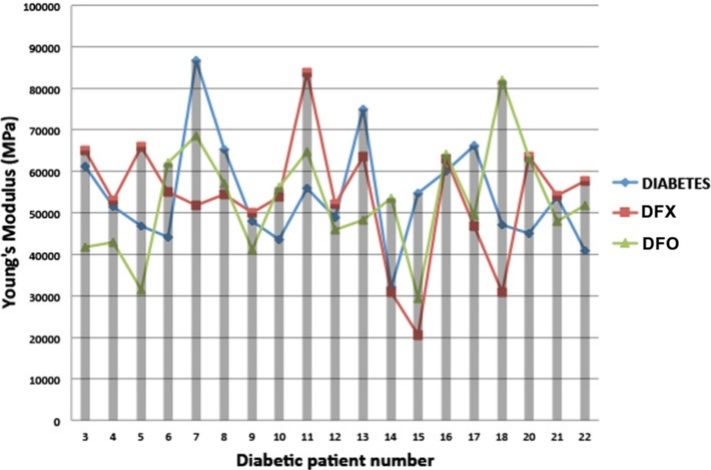
**Young’s modulus (stiffness) measurements to show trends in 19 of the individual diabetic erythrocytes after treatment with DFX and DFO.**

However, when the elasticity measurements are compared individually after treatment with the iron chelating agents a more complex picture emerges, due to the substantial variation in the two cohorts. Although on average the iron chelators caused a significant increase in elasticity, more than 50% of the diabetic group treated with DFX and almost 40% treated with DFO showed an increase in the elastic (see Figure 8).

### Scanning electron microscopy of fibrin network

**Figure 9:**
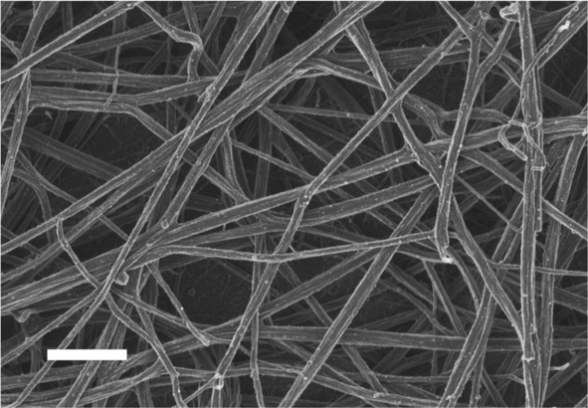
**Plasma from a healthy individual, with added thrombin, to create an extensive fibrin network.** Male: 58; serum ferritin: 208 ng.mL^−1^. Scale Bar = 1 μM.

**Figure 10:**
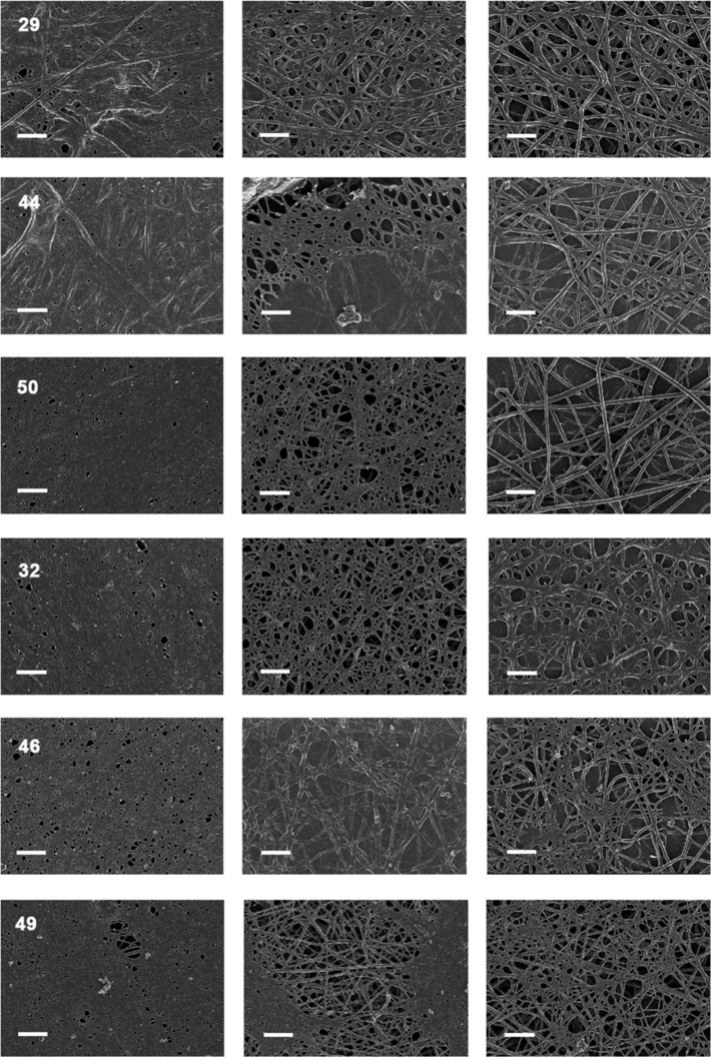
**Fibrin formed in plasma from diabetic patients before and after treatment.** Left: untreated; middle: Deferasirox (DFX) and right: desferrioxamine (DFO).

Figure 9 shows plasma with added thrombin to create an extensive fibrin network from a typical healthy individual. Figure 10 shows whole blood from diabetic patients before and after treatment, where column A shows untreated WB, column B shows WB treated with DFX and column C shows WB treated with DFO. Smears were chosen that represents the sample, e.g. normal iron levels, increased iron levels and whether addition of chelators showed a significant P-value (or not); see Table 7 for specific details of the patients shown in the figure. Typically, fibrin forms individually visible fibres. However, in the presence of inflammation, the fibrin clots abnormally, to form finer fibres and in some cases, a continuous layer, where individual fibres are not visible. This has previously been noted in diabetic patients [95,96]. With the addition of the 2 chelators, fibrin nets with visible individual fibres are seen. This was found irrespective of increased SF levels (see Table 9) and we have argued [139] that serum ferritin – which not even exist as it is it not an extracellular iron transporter – is probably devoid of much iron anyway.

**Table 9:** **Diabetic patient details of micrographs from the SEM analysis shown in Figure** **10**

	**Gender**	**Age**	**Iron (μmol.L** ^**−1**^ **) 11.6-31.3**	**Transferrin ** **(g.L** ^**−1**^ **) 2.2-3.7**	**% saturation 20 – 50%**	**Serum ferritin (ng.mL** ^**−1**^ **) M = 20-250 F = 10-120**
**DIABETES 29**	M	72	25.3	2.4	42	**386**
**DIABETES 44**	F	66	28.6	2.6	44	**359**
**DIABETES 50**	M	61	23	***1.9***	48	**1097**
**DIABETES 32**	M	55	**10.3**	2.2	***19***	88
**DIABETES 46**	F	69	***9.6***	2.3	***17***	74
**DIABETES 49**	F	62	12.4	2.2	23	58

Bold values are above and italic values below the reference (normal) range.

## Discussion

Type II diabetes mellitus is a metabolic disorder of deranged fat, protein and carbohydrate metabolism resulting in hyperglycaemia from insulin resistance and inadequate insulin secretion [152]. This condition is very difficult to treat and even in countries like the USA, fewer than 50% of patients achieve the HbA1c goal of < 7% set by the American Diabetes Association [152,153]. One of the reasons might be poor adherence to treatment regimes [154-156], as well as poor diet and knowledge regarding nutrition [157].

In the current work, we noted a changed RBC shape and membrane structure, as well as a matted fibrin fibre structure. Previously, we suggested that this might be due to oxidative stress and increased iron levels [93]. Oxidative stress in diabetic subjects, including those with cardiovascular manifestations, may also be attributed to the hyperglycaemia which modifies the RBC membrane dynamic and electrokinetic properties when compared to healthy controls [158].

In the present study, we noted that the axial ratio of the RBCs from diabetic patients was significantly greater than that of matched controls, as seems to occur in a variety of inflammatory diseases [140,143,159]. Iron dysregulation is often intimately involved and unliganded iron can act in one of two main ways, viz simply by electrostatics or via its ability to catalyse hydroxyl radical formation with covalent modification (by hydroxyl radicals) of proteins and other macromolecules [139,141,160]. The former, but not the latter, may be reversed by iron chelators [139]. In the present study, the chelators had no effect on the axial ratios, consistent with the view that in the chronic conditions of diabetes the changes are mainly due to hydroxyl radical formation. However, the chelators did have effects on the detailed morphology of the RBCs, suggesting some contribution of electrostatic forces. AFM measurements indicated that the membrane stiffness of the RBCs of diabetics was significantly greater than that of controls, and that overall this could be alleviated by the addition of iron chelators. However, a detailed analysis showed that this was quite patient-specific.

Recently, Berndt-Zipfel and coworkers, showed that RBC deformability (measured using a laser-assisted optical rotational cell analyzer by determining the elongation index) was also changed in type II diabetes, and that an improved RBC deformability correlate of improved glycaemic control [105]. The current patient sample mostly had very poor glycaemic control, and this might partly be the reason for limited RBC shape and deformability improvement.

In a similar vein, the ultrastructure of the fibrin clots formed in the blood of diabetic patients following thrombin addition *in vitro* were highly aberrant in nature relative to those from the controls, much as we have seen before in a variety of inflammatory diseases. In this study, the chelators could act to make the fibrin morphology much more like those of the controls, consistent with the view that at least of these effects were due to the presence of unliganded iron acting electrostatically to modify fibrinogen and its behavior during polymerization to form fibrin.

Overall, we note, as we have before in a number of inflammatory diseases [139,141,143,159,160] that patients with type II diabetes manifest this via significant changes in both the morphology of their erythrocytes and in the nature of the fibrin fibres formed upon the addition of thrombin. We do not yet know whether these morphological changes have prognostic (as well as diagnostic) significance, but it is clear that lowering the amounts of labile iron are likely to be of benefit, as serum ferritin is an important disease marker, and implicated in most inflammatory conditions. Previously, we argued that and it is mainly a leakage product from damaged cells [139]. There are therefore possible clinical implications for “labile” (chelatable) iron in the thrombin-elicited fibrin formation in the plasma of diabetic patients, and also the elasticity of RBCs, possibly associated with serum proteins.
